# Realist Synthesis of the International Theory and Evidence on Strategies to Improve Childhood Vaccination in Low- and Middle-Income Countries: Developing Strategies for the Nigerian Healthcare System

**DOI:** 10.15171/ijhpm.2019.120

**Published:** 2019-12-17

**Authors:** Oluwadamilola Solabi Omoniyi, Iestyn Williams

**Affiliations:** Health Services Management Centre, University of Birmingham, Birmingham, UK.

**Keywords:** Realist Synthesis, Vaccination, Immunisation, Nigeria, Low- and Middle-Income Countries

## Abstract

**Background:** Childhood vaccination coverage rates in low- and middle-income countries (LMICs) vary significantly, with some countries achieving higher rates than others. Several attempts have been made in Nigeria to achieve universal vaccination coverage but with limited success. This study aimed to analyse strategies used to improve childhood vaccine access and uptake in LMICs in order to inform strategy development for the Nigerian healthcare system.

**Methods:** A realist synthesis approach was adopted in order to elucidate the contexts and mechanisms wherewith these strategies achieved their aim (or not). Nine databases were searched for relevant articles and 27 articles were included in the study. Programme theories were generated from the included articles, and data extraction was carried out paying particular attention to context, mechanism and outcomes configurations.

**Results:** Interventions used in LMICs to improve vaccination coverage were categorised as follows: communication/ educational, reminder-type, incentives, social mobilisation, provider-directed strategies, health service integration and multi-pronged strategies. The strategies that appeared most likely to be effective in the health contexts of contemporary Nigeria include communication and educational interventions; employing informal change agents, and; monitoring and evaluation to strengthen communication. The programme theories for the use of reminders, social mobilisation, staff training and supportive supervision were observed in practice, and these strategies were generally successful within some contexts. By contrast, the use of monetary incentives in Nigeria is not supported by the evidence, although further research and evaluation is required. The integration of other interventions with routine immunisation (RI) to improve uptake was more effective when the perceived value of the other program was high. Adoption of multipronged interventions for hard to reach communities was beneficial. However, caution should be exercised because of varying levels of published evidence in respect of each intervention type and a relative lack of the rich description required to conduct a full realist analysis.

**Conclusion:** This paper adds to the evidence base on the adaption of strategies to improve vaccine access and uptake to the context of LMICs.

## Background

Countries aim to achieve universal childhood vaccination for all eligible citizens due to the benefits associated with vaccination, such as prevention of infectious diseases related morbidities and mortality, improved health of individuals and reduced disease transmission within communities.^1^ However, in some low- and middle-income countries (LMICs), this goal has remained elusive. In 2017, World Health Organization Regional Office for Africa (WHO AFRO) region country leaders endorsed the Addis Declaration on Immunisation which focuses on 3 key aspirations: to generate and sustain political commitment and funding; strengthen technical capacity and overcome barriers to access, and; closely monitor progress.^2^ The aim was to address immunisation barriers in WHO AFRO countries documented in the Regional Strategic Plan for Immunisation 2014-2020 which include: suboptimal national ownership of immunisation programs; inadequate training of healthcare workers; inequities in access, and; inadequate community engagement.^3^ Overall immunisation coverage rates are typically measured as the proportion of infants who have received their third dose of diphtheria, tetanus and pertussis-containing vaccine (DTP3).^2^ DTP3 is used as a proxy to measure immunisation performance globally,^4^ in Nigeria and also used in this study. Based on the WHO country categorisation on the immunisation maturity grid, Nigeria is in category 2, with significant deficiencies in immunisation delivery (compared to countries in category 4 deemed to have robust immunisation systems).^3^ Vaccination coverage in Nigeria as at 2017 was 42%.^5^

Vaccination coverage in Nigeria has ranged between 21% in 1997 and 63% in 2009.^5^ After the peak coverage in 2009, there was a gradual decline and the present estimated DTP3 coverage of 42% has remained stagnant since 2015 to 2017.^5^ This is significantly lower than the global average of 86% and the Nigerian government’s target of 95% by 2020.^4,6^ Reasons for the poor performance in vaccination coverage in Nigeria have been attributed to factors such as inadequate skilled human resources in rural areas, poor coordination between immunisation program non-governmental organisations and the National Expanded Programme on Immunisation (EPI), inadequate funding, and waning support of some political leaders.^6,7^

Multiple strategies have been used to improve national immunisation coverage in Nigeria. These include the comprehensive multi-year plan 2011–2015 which aligned with the Global Immunisation Vision and Strategies, and the National Routine Immunisation Strategic Plan 2013– 2015.^8,9^ Comprehensive multi-year plan promoted routine immunisation (RI) provision in health facilities through: local immunisation days and the Child Health Week which involved community-based immunisation campaigns; the Reaching Every Ward strategy; data management and monitoring systems; vaccine supply and quality initiatives, and the cold chain system.^8^ The National Routine Immunisation Strategic Plan’s focus was on logistics, service delivery, supportive supervision, health management information systems, community participation and ownership, leadership and governance, partnerships and programme integration, and research for RI.^9^

The majority of the strategies outlined in the current Nigeria Strategy on Immunisation and Primary Health Care Systems Strengthening (NSIPSS) 2018‒2028,^10^ are similar to past strategic plans developed at the country level addressing issues such as building capacity of health workers, strengthening data systems, improving service delivery at primary healthcare and outreach sites, and improving cold chain systems, vaccine availability and demand creation.

The low vaccination coverage in Nigeria despite all of these strategic plans portends the need to develop better implementation approaches by learning from in-country experiences and international evidence, in order to achieve universal vaccination coverage amongst Nigerian children.

### LMIC Vaccination Statistics

According to the World Bank classification,^11^ LMICs are countries with 2018 gross national income (GNI) per capita of $12 375 or less. This ranges from low income countries to upper middle-income countries. Nigeria with a GNI per capita of $1960 is categorised as a lower middle-income country.^11^ In 2017, the average DTP3 immunisation coverage in LMICs by age 12-23 months was 84.5%.^5^ Despite this seemingly acceptable average vaccination rate, the majority of children who die from vaccine preventable diseases are from LMICs where there is a wide variation in vaccination coverage, ranging from 25% in Equatorial Guinea to 99% in Ghana. Nigeria lies in the lower half with DTP3 coverage of 42% in 2017.^5^ Equatorial Guinea with the lowest DTP3 coverage in 2017 is an upper middle income country, while Rwanda with one of the highest DTP3 vaccination rates (98%) is a low income country.^5,11^ Therefore, Nigeria’s GNI per capita cannot serve as an explanation for its low vaccination coverage.

The socio-economic context in Nigeria, as in a significant number of LMICs, includes a high level of poverty, unemployment and illiteracy with geographical variation across the country.^12^ These have a strong influence on immunisation uptake.^13^ Also, Nigeria has a large rural community with hard to reach groups such as the nomadic communities and those living in riverine areas. This is similar to India, which also has communities living in hard to reach riverine areas for which boat clinics were devised to provide primary healthcare.^14^

Many LMICs have contextual features that are comparable to the Nigerian context as described above, and a significant number of them have achieved high vaccination rates. The aim of this realist synthesis is therefore to develop a deeper understanding of which interventions used in LMICs have been successful at improving vaccination coverage, and also to shed light on how, why and in what circumstances they worked. In addition, the review will provide information on the mechanisms of success of a broad range of intervention types and help distinguish between those with good supporting evidence, and those requiring further research. Furthermore, documenting the contextual factors will increase understanding of the external validity of the interventions, and therefore inform their reproducibility in other settings.

Learning from effective vaccination strategies in LMICs, and adapting them to the Nigerian context, is pivotal to achieving universal childhood immunisation in Nigeria as this may ultimately reduce unnecessary vaccine preventable morbidities and mortalities. Therefore, this research aims to contribute towards the theory and evidence base which can be drawn upon to develop Nigeria’s immunisation policies and practice.

## Methods

Realist synthesis involves the systematic review of primary studies in order to identify, test and refine programme theories, thereby providing insight for possible transferability of interventions across borders.^15,16^ Realist synthesis was deemed suited to this research because it enables elucidation of the factors and contexts that facilitate or hinder the implementation and impact of strategies to improve vaccination coverage. Also, it provides explanations for why the strategies work and for whom, in what settings. There is no single, accepted definition of ‘context’ but a helpful distinction has been made between ‘inner’ and ‘outer’ context.^17^ In this review we were interested in, for example, organisational structures and cultures (inner) and wider system, economic, social and political factors (outer).

Although a previous systematic review by Oyo-Ita et al had focused on interventions for increasing child immunisation coverage in LMICs, this provided only very limited insight into aspects of context and programme theory.^18^ This limits its efficacy in informing immunisation policy formation and implementation. This study aimed to overcome this limitation through the use of realist synthesis which generates explanations for how and in what circumstances interventions worked during the process of synthesis, thereby increasing understanding of transferability to other contexts.

To carry out a realist synthesis, programme theories are first generated from a scoping search of the literature, and then empirical evidence is interrogated to determine if they are manifest in practice.^19^ The intention is to create a revised model of how and why programmes work which then can be used to provide advice on introduction and implementation of interventions.^19,20^ In this review, six steps were followed, as outlined below^15,19^:

1. Clarification of review question.
2. Search for primary studies: first, a scoping search was conducted to ascertain the availability of published materials on LMIC immunisation maximisation strategies. Then, a full search was carried out in order to generate programme theories and collate empirical evidence to test the theories.
3. Appraisal: this involved assessing each primary study for relevance in order to determine whether it has the appropriate content to contribute to the review. For the quality appraisal, 2 main questions were applied, and articles were included if the answer to both questions was yes:
• Did the authors refer to patient/community-oriented, provider-directed or health system-directed interventions to improve vaccination rate in their paper?
• Did the paper provide relevant contextual details required for realist synthesis?
4. Extracting the data: first, annotation of relevant texts was carried out, followed by note-taking on programme theories. Then, collation of materials from selected primary studies, and detailed extracts from each case study were undertaken. A data extraction form was used to collect descriptive and substantive information from each included item.
5. Synthesizing the data: this involved considering the same theory in comparable settings in order to produce a revised general theory of conditions that support or hinder the programme theory, and identifying any weak points.
6. Disseminating the findings.

The principles of PRISMA^21^ were followed in this study although specifically, realist approach^15^ was used to guide synthesis (Figure). Database searches were conducted in July 2016. To ensure relevance of included studies we set a time limit for inclusion of 20 years so that all studies prior to 1996 were excluded.

**Figure:**
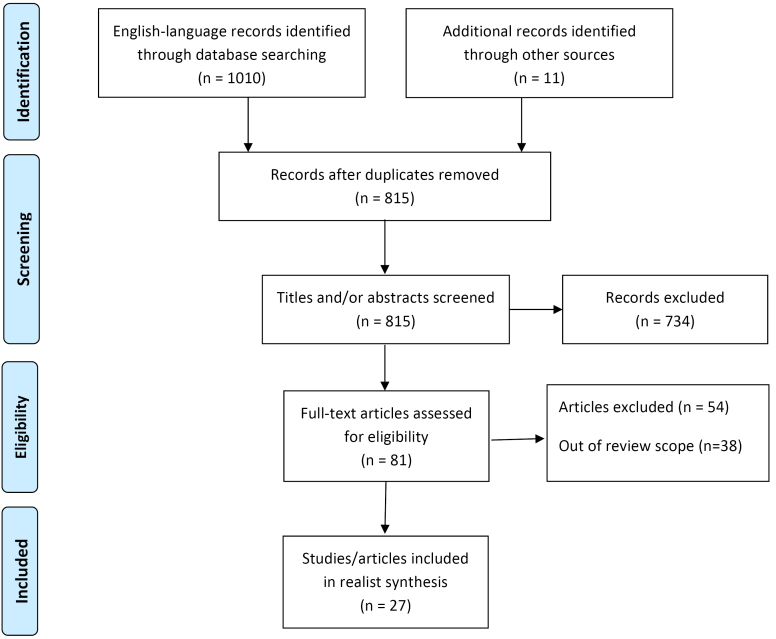
PRISMA Flow Diagram.

### Inclusion Criteria

- Primary studies including randomised controlled trials, non-randomised controlled trials, controlled and uncontrolled before-and-after studies, cohort studies, case-control studies, cross-sectional studies and qualitative evaluations.
- Studies involving children under 5 years of age, as most childhood vaccinations are given within this age bracket. In order to maximise relevance, included literature was drawn from LMIC countries.
- Studies on interventions used to improve vaccine coverage were included and grouped according to the following types:
- Patient or community oriented interventions such as communication/educational interventions; incentive-based interventions; and reminder-type interventions

- Provider-directed interventions
- Health service integration interventions
- Multi-pronged interventions which involves combinations of the above interventions

### Exclusion Criteria

- Non-empirical and grey literature
- Studies involving immunisation strategies in high-income countries
- Studies conducted before 1996
- Studies published in languages other than English (for reasons of time and resource limits)

### Search Strategy and Sources

Following initial scoping searches for relevant articles, a list of search terms and synonyms were developed and these were subsequently used in full database searches of: Health Management Information Consortium, EMBASE, Social Policy and Practice, Cochrane library, MEDLINE, Web of Science, International Bibliography of the Social Sciences, CINAHL, and Scopus. Searches were conducted using the following keywords (search terms):

‘Vaccination’ (vaccin*) OR ‘immunisation’ (immuni*) [Searched for in title or topic] AND ‘strategies’ (strateg*) OR ‘interventions’ (intervention$) OR ‘innovations’ (innovation$) OR ‘policies (polic*) OR ‘programmes’ (program*) [searched for in title or topic] AND ‘access’ OR ‘coverage’ OR ‘uptake’ OR ‘reach’ [searched for in title or topic] AND ‘childhood’ (child*) OR ‘infant’ (infan*) OR ‘baby’ (bab*) [searched for in the entire text].

The search strategy was adapted to suit each database using the applicable vocabulary. The titles and abstracts of the hits generated by the search strategy were screened for relevance. The inclusion criteria were applied to identify relevant articles which were subsequently included in the realist synthesis. Where previously conducted systematic reviews were identified, primary studies included in those systematic reviews were also retrieved. Hand-searching of bibliographies of relevant documents and supplemental online searches (eg, Google scholar) were carried out. Screening and study selection was conducted by the first author and checked by the second author. Assessment of relevance/conceptual richness was conducted independently by first and second authors with any discrepancies resolved through discussion. Search results were saved into Refworks reference manager and duplicates eliminated. Full texts of all potentially relevant articles were retrieved and screened through application of the exclusion criteria.

### Data Collection and Analysis

For all eligible studies, a structured data extraction form was used by the first author to record descriptive characteristics of each literature item and to record data on: underlying programme theories, contexts, mechanisms and outcomes. These were checked by the second author. Data analysis was conducted manually as an iterative process in line with the research question and objectives of the study. Where 2 articles reported the same study, they were merged. The studies were assessed for conceptual richness using the framework by Pearson et al,^22^ and subsequently divided into 3 categories: conceptually rich; thicker description, and; thinner description. Conceptually rich articles have unambiguous, well-grounded and sufficiently defined theoretical concepts, and well-elucidated relationships between and among concepts. Articles with thicker description provide enough description to enable the programme theory be surfaced, consider the programme’s context, discuss the differences between programme theory and implementation, the strengths and weaknesses of the programme as implemented, and attempt to explain anomalous results. Lastly, articles with thinner description give only basic information on programme theory and context, provide little or no discussion on the differences between programme theory and implementation, strengths and weaknesses of the programme and factors affecting implementation.

The main outcome considered in the review was increase in overall rates of vaccine coverage, but it also included increased coverage in specific groups. All of the included studies were operating with outcome measures relating to increased vaccine coverage.

Realist synthesis enables contextually-sensitive assessment of the effectiveness of interventions, which can be used to build middle-range theories about likely levels of effectiveness in other comparable settings. This was the underpinning basis for making recommendations for future strategies to improve vaccine coverage in Nigeria.

## Results

The evidence base used in this review was generated from interventions used in LMICs across different world regions as follows sub-Saharan Africa (8), Asia (13), North America (3), Middle East (2) and 1 cross-country study in sub-Saharan Africa and Asia. Studies included randomised control trials, controlled before and after studies and qualitative evaluations. These included 12 studies conducted in rural settings, 11 in urban settings and 4 in rural and urban settings. Three studies addressed hard-to-reach communities in LMICs. Seventeen of the 27 included studies, evaluated interventions directed at individuals and families within communities, 2 were directed at health workers, 4 involved integration of other interventions with RI, and the remaining 4 involved multi-pronged interventions addressing both the supply and demand sides of vaccination. Of the 17 studies directed at individuals and communities, nine addressed communication/educational interventions including 2 studies which combined reminder-type interventions with communication; 2 others addressed parental reminders only; 4 studies addressed the use of incentives to improve vaccination uptake; and 2 studies addressed social/community mobilisation. Supplementary file 1 contains a description of articles included in the realist synthesis, categorised according to their intervention type, with brief descriptions of settings, participants, methods and outcomes of each intervention.

To elucidate the programme theories underlying interventions, the studies were divided into seven intervention categories as follows: Communication/educational; incentive-based; reminder-type; community or social mobilisation; provider-directed; health service integration; and multi-pronged strategies. Table contains a description of the programme theories underlying each intervention category, drawing on information provided by all studies included within each category.

**Table:** Programme Theories Underlying Intervention Categories

**Intervention Type**	**Sources**	**Programme Theory**
Communication/Educational	Andersson et al 2009^23^; Bolam et al 1998^24^; Oku et al 2016^25^; Owais et al 2011^26^; Pandey et al 2007^27^; Abdul Rahman et al 2013^28^; Usman et al 2009^29^; Usman et al 2011^30^; Waisbord et al 2010^31^	Low vaccine uptake is believed to be a major causative factor of reduced vaccination rates. Lack of awareness and false beliefs within populations are believed to negatively impact vaccine uptake. Hence, it is believed that effective communication and education will assist in raising awareness; creating and sustaining demand; preventing or dispelling misinformation and doubts; encouraging acceptance of and participation in vaccination services; and more rapid reporting of disease cases and outbreaks. It is assumed that having the right information will result in making rational decisions and following through with appropriate action. Also, it is believed that communication will inform people about where and when to get immunised, and thereby increase vaccination rates. Some researchers propose that educational interventions should aim to provide information on the cost-benefits of vaccination compared with treatment of vaccine-preventable diseases, because this will motivate people to vaccinate their children. The focus of some authors is on the duration and amount of information passed in each educational session, and they believe sessions with shorter duration and more focused content will produce better retention and behaviour modification. Also, some believe that communication will work through the use of influential persons in communities to pass across vaccination messages; one-to-one sessions with mothers and providing information on entitled services.
Incentive-based	Banerjee et al 2010^32^; Barham and Maluccio 2009^33^; Maluccio and Flores 2004^34^; Morris et al 2004^35^; Robertson et al 2013^36^	Incentives are believed to motivate people to carry out actions. Incentives work through external motivation according to the theory of motivation. It is believed monetary incentives will raise awareness about beneficial behaviour, and enable people make the right choices by covering the financial and opportunity costs that would otherwise have accrued to them and prevented vaccination uptake. Some authors believe adding conditions to these monetary transfers will ensure compliance and result in immunisation completion. Conditional cash transfers are supposed to act as human capital subsidies for poor households, which would enable them invest in the health and education of their children. Also, some believe that non-monetary incentives such as raw lentils and metal plates will provide small benefits that might overcome little barriers that hold the key to large improvements in immunisation rates.
Reminder-type	Bangure et al 2013^37^; Domek et al 2016^38^; Usman et al 2009^29^; Usman et al 2011^30^	The advocacy of parental reminders assumes the reason for reduced uptake is forgetfulness, and that enhancing recall of immunisation appointment dates, times and venues would increase uptake. It considers reminders to be a valid mechanism for communication between parents and healthcare providers which can be harnessed to educate parents on the importance of vaccine completion, and encourage them to return for their vaccination appointments, thereby sharing some underlying assumptions of ‘communication/education’ interventions.
Community/Social mobilisation	Brugha and Kevany 1996^39^; Weiss et al 2011^40^	It is believed that social mobilisation efforts addressed to the grassroots will reach underserved populations through Supplementary Immunisation Activities to reach them at the community level, and will combat rumors against vaccination. Also, home visits will enable eligible children be identified and referred for immunisation, and pockets of low coverage will be identified and addressed. In addition, it is assumed that any intervention in peoples' homes that is tailored to meet their needs, if implemented in a sensitive way is likely to have a positive impact.
Provider-directed strategies	Djibuti et al 2009^41^; Uskun et al 2008^42^	These strategies assume that bottlenecks lie principally with those charged with provision of vaccines. Therefore, supportive supervision will enable staff to carry out their duties effectively by providing guidance, support, motivation and assisting staff to become more competent in their work. Also, staff training will improve immunisation knowledge and skill amongst staff, and thereby reducing missed opportunities and drop-outs.
Health service integration	Briere et al 2012^43^; Dicko et al 2011^44^; Mathanga et al 2009^45^; Ryman et al 2012^46^	The basis for this intervention is that RI programmes have the greatest and most equitable coverage of all childhood preventive programmes in the developing world, and also provide multiple health contacts with mothers and their children. Hence, the reach of other health interventions can be extended by integrating them with RI. Also, the availability of other health interventions such as hygiene kits or insecticide treated nets will act as incentives to increase vaccination coverage.
Multi-pronged strategies	Hayford et al^47^; Hu et al^48^; Uddin et al^49^; Uddin et al^50^	The multi-pronged programmatic approach is believed to pull together the benefits of different proven interventions that address both the demand and supply aspects of the vaccination coverage problem, in order to produce a complete package that can improve immunisation coverage because it is believed that singular interventions are not sufficient to improve vaccination coverage, especially in hard to reach communities.

Abbreviation: RI, routine immunisation.

### Programme Theories Underlying Intervention Categories

Programme theories are statements used to describe the underlying assumptions upon which programmes are built, by explaining why, how, and under what conditions programme effects occur.^51,52^ Interventions that seek to improve vaccination coverage usually address: the demand side of vaccination uptake directed at individuals, families or communities; the supply side of vaccination access aimed at healthcare workers; health systems strengthening, or; a combination of interventions.^53^

Demand side strategies are usually based on the assumption that identifiable factors such as ignorance, financial constraint and poor recall of vaccination schedule, influence non-uptake of vaccination.^25,31-33,38^ Conversely, supply-side strategies are based on the assumption that health workers’ lack of knowledge and skills, poor supportive supervision and lack of motivation are the main factors affecting the quality of care provided to children resulting in low vaccination coverage.^34,41,42^ Hence, strategies are developed based on the assumption that resolution of these factors will improve vaccination rates.

###  Results of the Realist Synthesis of Empirical Evidence

A realist synthesis was conducted using the programme theories to trace interactions between context, mechanisms and outcomes configurations of each of the strategies. This section is divided into seven subsections, each of which addresses the intervention categories described above.

### Communication/Educational Interventions

Communication and educational interventions were successful within a variety of contexts. These interventions seek to improve vaccination uptake by providing parents and caregivers with information on the benefits of vaccination, and the risks of non-vaccination or incomplete vaccination.^29^ This is because it is believed that awareness would result in behavioural change, thereby increasing vaccine uptake and reducing dropouts.^29^ This programme theory worked via various mechanisms in different contexts. In Bauchi State Nigeria, Iraq, Pakistan, and Afghanistan, which are predominantly traditional Muslim societies, the education and engagement of traditional and religious leaders as advocates for immunisation helped enhance communication with the community, thereby increasing acceptance of vaccination.^25,28,31^

In some settings, religious leaders enjoy legitimacy that political leaders do not have. Hence they can contribute towards an improvement in vaccination coverage by counteracting negative rumours about vaccination.^31^ Similarly, Pandey et al in India demonstrated that the absence of leadership involvement hindered uptake.^27^ The mechanism in this case therefore appears to relate to the credibility of the source of the information provided. Furthermore, communication strategies worked by reviewing the content of educational sessions to include enlightenment of communities regarding their health entitlements such as free vaccinations. It worked in the context of a community in rural India that was plagued with high levels of absenteeism of healthcare workers. The increased awareness had an impact on the demand and supply sides of vaccination. However, the use of that particular strategy in isolation may not produce the desired impact because other factors such as transportation and opportunity costs have been shown to prevent access levels among poorer families even where awareness levels are high.^35,36^

Andersson et al studied poor communities in Pakistan and proposed that discussions around the costs and benefits of vaccination using accurate local information can shift the scale favourably towards vaccination uptake in poor localities.^23^ However, the counter-argument was raised that local remedies for illnesses such as measles are cheaper (albeit less effective) than vaccinating the child.^23^ This shows that educational programmes need to critically appraise the communities they are addressing and review local interventions and culture before proposing what may be perceived as alien practices. Also, other unanticipated mechanisms may arise during the implementation of a programme. Andersson et al report that mechanisms like carpooling, which reduced the transportation costs to vaccination points, and providing care for children while parents took others to be vaccinated, emerged in the course of their discussions.^23^ This demonstrates the importance of allowing other strategies to be identified during discussions, because these may surface the actual concerns of the caregivers.

Furthermore, on the content and duration of educational sessions, Owais et al in Pakistan demonstrate that vaccine completion rates were significantly higher in children of mothers that received focused, short duration (5 minutes) education on immunisation, rather than longer (10-15 minutes) general health promotion messages which included information on vaccination.^26^ Effective mechanisms include the use of pictorial messages and home visits for these focused educational sessions. According to Oku et al, in Nigeria, targeting caregivers at home through home visits, radio and television messages, enabled access to, for example, some Muslim women who would not leave their homes due to the Purdah system.^25^ Also, Bolam et al’s finding in Nepal buttressed the point that using long duration general health education sessions may be ineffective due to information overload and reduced focus on immunisation.^24^

###  Reminder-Type Interventions

The use of reminders to increase vaccination uptake is based on the assumption that forgetting immunisation dates, times and locations is a major factor contributing to reduced vaccine uptake. In Zimbabwe, Bangure et al^37^ studied the effect of SMS reminders on vaccination where each household had at least one functional mobile phone, and the text messages were sent in the local language. They reported significantly higher vaccination coverage and reduced delays in the intervention group compared with controls. On the other hand, Domek et al^38^ found different results for a similar study in Guatemala. In this case, both the intervention group and usual care group had high vaccination completion rates and the difference was not statistically significant. This may be because the children presented for their first vaccination on time prior to intervention and were recruited into both groups at that time resulting in selection bias. Furthermore, they experienced some power outages which affected the delivery of text messages and this may have contributed to the insignificant difference between the groups. SMS messages were judged to be cost-effective in both studies, and well-received by the intervention group in the low resource setting compared with the control group. This may be because the control group participants were able to achieve high vaccination rates without SMS reminders, so they may not deem the extra cost to be warranted.

However, a more cost-effective means of reminder utilised in Pakistan, involved the use of redesigned reminder-type immunisation cards which showed only the next immunisation date and day on both outer sides in large font and was attached to a hanging string; this enabled them to be easily displayed in conspicuous places in the home. The remaining client information was written inside the folded card and the previous appointments were struck off thereby focusing attention on the next appointment.^29,30^ This intervention was combined with brief centre-based maternal education during each immunisation visit. In the rural setting, the interventions were successful in increasing follow up immunisation visits either singly or in combination. However, the use of the redesigned cards alone was as effective as the combination of both interventions, and more effective than education alone. Findings in urban settings also demonstrated the impact of the redesigned card. Both settings were characterized by low literacy levels which suggest that such a low cost intervention may be useful in similar settings in other countries. Also, in the urban setting, most of the people attending the EPI centres are lower and middle socio-economic class citizens, so such a cost-effective strategy would be beneficial to them.

### Incentive-Based Interventions

In all of the included studies on the use of monetary incentives, the aim was to improve the use of a range of health and social services, not vaccination alone. This is most likely due to financial limitations which would prevent each health intervention from having its own separate monetary incentive. Also, these incentive-based interventions were conducted in poor communities so that the impact of the incentive will be of material significance and interest in the health intervention sustained. A common theme across majority of the included incentive-based studies was that the effect could not be clearly attributed to the incentives because the control group also demonstrated improvement, or alternatively that there was an improvement which was either not sustained or was insufficient to attain desired vaccine coverage levels.

In Zimbabwe, neither unconditional nor conditional cash transfers significantly increased the number of children with complete vaccination records.^36^ This may be because vaccinations were often delivered via mobile outreach and cash transfers did not affect access to these services. Also, the conditions for conditional cash transfers were not always enforced, hence households received their cash transfers irrespective of whether they met them. Therefore, the programme theory was not manifest in practice because the separable outcome (cash transfer) did not hinge on performance of the activity (vaccination uptake).

However, in rural Nicaragua, following the introduction of conditional cash transfer, immunisation rates became significantly higher in the intervention than control group, with larger effects in hard to reach communities.^33,34^ This success could arguably be attributed to other mechanisms incorporated into these programmes: high levels of planning and coordination; community involvement; utilisation of mothers as household representatives; transparency in the group selection process, and; the use of both demand and supply side strategies. However, similar to the Zimbabwean study, there was a substantial increase in vaccination coverage in the control group, possibly because the conditions in the intervention group were not strictly adhered to and the Ministry of Health operations in the control localities were strengthened during the study period.^33^ The increase in vaccine coverage in both intervention and control sites makes it difficult to attribute the success achieved solely to the intervention.

In rural Honduras, where monetary incentives were directed at households and/or health services, there were difficulties implementing the health service package but the household packages were implemented as planned.^35^ The outcome was an unsustained increase in vaccination rates. The authors did not clearly explain this finding but it can be inferred that since documentation shows that the mothers regularly visited the health facilities for growth monitoring, but the vaccination completion rate did not improve, then the missed opportunities were a service delivery problem. This indicates that addressing one part of the equation (demand) while leaving the other (supply) unattended may affect outcomes.

Banerjee et al argue that incentives work irrespective of their magnitude.^32^ Their study in rural communities in India, which involved the use of lentils and metal plates as non-monetary incentives, demonstrated an increase in full vaccination rate from 2% to 39% in those who received incentives and improved service, 18% in those who received reliable immunisation without incentives, and 6% in control villages. Despite the significant improvements in the first group, these rates are still too low to achieve herd immunity.^32^ This raises the question of whether more substantial incentives might lead to greater gains in vaccine coverage. However, this clearly has cost implications and most of the articles on incentive-based strategies did not rigorously model cost and benefits. Further inquiry is therefore required in this area.

### Social/Community Mobilisation

Social mobilisation at the community (or ‘grassroots’) levels aim to reach underserved populations with supplementary immunisation activities at or close to their homes by vaccinating eligible children within their communities. These interventions engaged non-health workers (NHWs) such as female community mobilisation coordinators and school children to reach mothers within communities resistant to vaccination, identify eligible children and refer them for vaccination. Also, direct personal communication with community leaders and families through home visits to dispel myths, educate mothers and vaccinate children resulted in higher immunisation uptake.^39-40^ This suggests that NHWs in collaboration with healthcare providers have an important role to play in improving vaccination coverage. Also, it can be argued that the convenience and efficiency of having the vaccines administered at home, and the involvement of fathers in the decision to vaccinate their children, contributed to the gains.

The next section will address the strategies directed at improving immunisation provider knowledge and competence.

### Provider-Directed Strategies

In Turkey, health worker training led to increased vaccination rates and reductions in missed opportunities.^42^ Also, data recording and reporting skills increased. The content of the training and performance of trainers had an influence on effectiveness of the intervention. The fact that all vaccines are provided free may also have helped because the cost of vaccine did not hinder its administration.

In Georgia, within the context of health reforms with increased health system financing and poor health worker performance, supportive supervision worked by improving provider knowledge and skills, and increasing communication between supervisors and staff, leading to prompt addressing of day-to-day challenges.^41^ Hence, the intervention contributed to reduction in vaccine wastage and increased DTP3 immunisation coverage.

### Health Service Integration

The majority of the strategies to integrate other interventions with immunisation programme were based on the premise that immunisation programmes have the most equitable distribution of all childhood prevention programmes and also provide several opportunities for contact with children. Hence the focus of these integration programmes was to improve the coverage of other interventions by integrating them with immunisation, and either maintain or increase immunisation rates in the process. Briere et al in Kenya reported an increase in hygienic practices following integration,^43^ but the effect on immunisation was unclear because both intervention and control sites demonstrated increases in immunisation, albeit varying across rural and urban communities. Ryman et al reported similar findings in their Kenyan study which showed increased vaccination rates in urban areas but static or reduced rates in rural areas.^46^ Distances to hospital, transportation costs and stock-outs were reported to be the major causes of unsuccessful vaccine coverage in rural areas. Conversely, Mathanga et al in Malawi,^45^ reported an appreciable increase in timely immunisation coverage and ITN utilisation. However, timely immunisation increased in both intervention and control sites. The increase in the control sites may be attributable to the introduction of WHO’s Reaching Every District strategy which involved vaccination programme planning and monitoring strategies in that district during the study period. This makes it difficult to attribute the gains made in the intervention sites to the integration programme.

In Mali Dicko et al attributed the significant increase in immunisation rates and Intermittent Preventive Treatment of malaria uptake in the intervention group to people’s concerns about malaria, belief that the drug is an antipyretic (because fever is a common adverse effect of vaccination), and because it is free.^44^ Also, there was increased staff training and supervision.^44^ This suggests that for integration to be mutually beneficial to both programmes, the programme being hinged unto RI must also be able to stimulate public interest, otherwise, it could be counterproductive to the continued success of RI.

###  Multi-pronged Interventions

Most strategies to improve vaccination coverage amongst hard to reach communities utilise multi-pronged efforts to increase the probability of achieving the objective. In Dhaka city, Bangladesh, the majority of the population live in slum households and vaccination coverage was low despite advances in other parts of the country. A multi-dimensional immunisation package which included^49^: an extended EPI service schedule; training for service providers; a screening tool to identify immunisation needs among clinic attendants, and; an EPI support group for social mobilisation was introduced which is similar to that introduced in other hard to reach communities such as migrant children in China and children living in hoar and hilly areas in Bangladesh.^48,50^

These immunisation packages produced significant improvements in vaccination coverage in all categories of hard to reach children, as well as leading to reduced drop-out rates. All study authors claimed the implementation of the immunisation package to be cost-effective in their settings, primarily due to its implementation within existing health services with no additional costs incurred.^48-50^ However, Hayford et al tested this hypothesis in Bangladesh and found that additional costs were incurred from external management and supervision, training, coordination, uncompensated staff and clinic time, communications and supplies.^47^ These extra costs affected the sustainability of the programme as following the pilot intervention, none of the clinics or support groups were able to continue with the intervention without additional financial resources.^47,48^ External supervision was the most expensive element while the screening tool cost least. In the urban slums of Bangladesh, it was believed that the screening tool was least effective because it identified few children. However, this study was not powered to compare the effects of the interventions within the package.^49^ Conversely, the study conducted in the hoars and hilly regions in Bangladesh compared the screening tool with other interventions in the package and found the screening tool to be most effective.^50^ For multi-pronged immunisation packages to be utilised routinely in many settings, it appears necessary to identify which combination of interventions produces the greatest impact at the lowest cost, so that implementation can be sustainable in the long run.

## Discussion

Based on the evidence generated from the realist review, certain generalised deductions can be made as to the applicability of strategies for improving childhood vaccination coverage in LMICs. The results show that communication interventions are likely to be most effective when they: utilize influential leaders; include cost-benefit discussions, and; increase awareness of health entitlements. SMS reminders are likely to be successful in contexts with 100% mobile coverage where each household has a functional mobile phone, through text messages in the local language. Reminder-type immunisation cards appear to work well in rural and urban settings by increasing visibility of the next immunisation date. Incentive-based strategies appear to be most effective when targetting hard to reach and poorer communities. Provider-directed strategies appear to be effective in contexts with poor health worker performance and free vaccination at the point of delivery, and when they incorporate health worker training and supportive supervision. Integrating other health services with vaccination appears to work best in settings where the perceived value of the other service is high. Finally, multipronged interventions appear to be successful amongst hard to reach communities if and when they incorporate the package into already existing health services.

### The Nigerian Context and Applicability of Evidence

Nigeria is a highly populous country with major religious, cultural and socioeconomic differences across its population. Its citizens range from nomadic communities to hard-to-reach sedentary communities living in riverine areas, with many rural and urban communities in between. For a strategy to be successful within any community, it has to consider the community’s context in order to address its peculiar needs, and on a scale sufficient make an impact, as not all interventions will have the same effects in all settings. Strategies used in LMICs discussed in the previous sections of the paper, cover interventions addressing a wide range of communities with some similarities to Nigerian sub-populations. There also appear to be some commonalities in barriers to vaccination access and uptake across most LMICs including Nigeria.

In Nigeria, the reasons for persistent low immunisation coverage include inadequate human resources and inequitable distribution of health workers across rural and urban areas, inadequate funding from government allocation, security challenges especially in Northern Nigeria, poor data quality and so on.^6^ Previous interventions in Nigeria addressed both RI and immunisation campaigns using strategies such as multi-pronged programmes (eg, the Reaching Every Ward strategy), health system strengthening to improve logistics, supplies, data management and monitoring, increased health worker capacity, communications and so on.^6^ The barriers to successful implementation of these interventions have been linked to poor funding, lack of political will, inadequate human resources and a weak healthcare system amongst other causes,^6^ similar to other countries in the WHO/AFRO region.^3^

Some of the interventions from other LMICs differ from those already implemented in Nigeria. For example, the use of financial incentives; reminder-like immunisation cards; and integration of malaria and hygiene interventions with vaccination are not commonplace in Nigeria. Within the limits of the information available within those studies, strategies are proposed for the Nigerian Healthcare system in the next section.

### Strategies for Routine Immunisation

To address some of the challenges facing RI in Nigeria outlined above, strategies addressing communication/education of caregivers and communities; reminders; provider-directed strategies; and health system strengthening to improve vaccine supplies should be considered.

Effective communication strategies are presently being utilised in various states in Nigeria.^25^ However, for this intervention to be equitable and sustainable across all states, the present gains need to be scaled up and strengthened. This can be done by learning from other LMICs. Successful mechanisms utilised in communicating effectively with individuals and communities include the use of influential religious or traditional leaders to give legitimacy and credence to the message being relayed.^28^ Most communities in Nigeria have religious and traditional affiliations which have a strong influence on their decision-making processes, so utilising these leaders to promote immunisation and break negative barriers would be beneficial.

Also, providing educational sessions in environments where caregivers are comfortable, in a manner that aids retention and is accessible to them offers the potential for better results. For example, use of home visits, short duration, targeted sessions and group meetings as means of aiding communication may be beneficial.^26,39,40^ One-on-one sessions did not convincingly demonstrate strategic advantage over group meetings, contrary to underlying programme theory.^23,24^ Therefore, and considering the time-consuming nature of this intervention, further study is required to ascertain its benefits.

The content of the educational sessions is also important. The advantage of using focused immunisation messages over general health promotion messages was demonstrated by Bolam et al and Owais et al.^24,26^ This is because focused messages are believed to aid retention and recall. Therefore, in Nigeria where provision of integrated child health services is promoted, it may be more feasible to dedicate specific health visits to teaching about vaccination only, and assign other days to teaching other child health promotion strategies. Furthermore, since all vaccines within the EPI schedule are provided free of charge in all government hospitals in Nigeria, it is important to disseminate this information and also provide information about costs and benefits of vaccination.^23,27^ The cost-benefit profile of vaccination may reflect contextual differences in Nigeria, as was demonstrated for measles management in Pakistan due to its thriving traditional medicine sector.^23^

Lack of awareness of immunisation schedules, times and places is another reason cited for low vaccination coverage in Nigeria.^8^ The evidence suggests this problem may be alleviated by the use of SMS reminders and reminder-like immunisation cards.^29,30,37^ Due to the low-costs and effectiveness of reminder-like cards in rural and urban settings, it may be more feasible to adopt this method nationwide. While in communities that have constant mobile phone network, the option of SMS messages can be offered and tailored to each individual’s needs. These educational and reminder-type interventions can be implemented in line with Nigeria’s NSIPSS 2018-2028 Strategic action plan directed at using demand creation strategies to increase demand for immunisation.^10^

Capacity building is another important challenge in Nigeria that cannot be over-emphasised as it can help to prevent missed opportunities and drop-outs, improve health workers’ knowledge, attitude and practice, and improve data recording and reporting. Capacity building can be achieved through staff training and supportive supervision.^41,42^ Training content and trainer capability are important factors that affect the impact of the training.^42^ Therefore, regular “train the trainer” sessions apart from staff training sessions should be held. Also, it is important to provide support for supervisors in order to enable effective supervision. This aligns with the current Nigerian strategic plan to build capacity of health workers at all levels.^10^

The use of incentives has mixed results which contradicts the underlying programme theory which assumes external motivation will cause a significant difference between the intervention and control groups. However, negative or null findings may reflect implementation challenges faced by those programmes.^35,36^ The NSIPSS 2018-2028 action plan highlights the use of incentives funded by the private sector as part of their corporate social responsibility.^10^ The impact, cost-effectiveness and sustainability of utilising financial or non-financial incentives in RI require further evaluation in the early stages of introduction into RI in Nigeria because of its significant cost implications. Also, discontinuation of incentive-based programmes after commencement may have a negative impact if that was the primary driver of vaccine uptake.

The main objective of health integration strategies was to improve the coverage of other interventions by integrating them with RI. These were found to be beneficial to the other programmes but not always to RI. The integration of malaria preventive strategies appeared to be more effective than hygiene practices, possibly because malaria is a more pressing and high profile public health issue. This deviates from the programme theory which assumed hygiene kits would incentivise as well, and suggests that the perceived value of the programme/incentive plays a more significant role. It is important to consider these factors when implementing integration in Nigeria.

For hard to reach communities in Nigeria such as children living in nomadic communities, urban slums, riverine or rural areas which lack healthcare facilities, adoption of multi-pronged interventions, as utilised in Bangladesh and China,^48-50^ may be helpful. However, for this programme to be sustainable, it would need to be adapted to meet the population’s needs bearing in mind the cost-effectiveness profile of each intervention within the immunisation package.

### Strategies for Immunisation Campaigns

Immunisation campaigns involve intensified time-bound efforts to build on RI progress while addressing gaps, and also showcasing the value of vaccines for the health of children and communities. The majority of the interventions discussed above for RI may be beneficial in immunisation campaigns also. As with RI, engaging influential leaders to legitimize and promote the vaccination agenda during immunisation campaigns, and providing focused immunisation messages in caregiver environment may be beneficial for increasing vaccination uptake during immunisation campaigns.^26,28^

Specifically for campaigns, communication can be enhanced through the use of town hall meetings, town criers, television announcements, radio jingles, hand bills and posters. Also, real-time monitoring and evaluation may aid in increasing the effectiveness of communication strategies.^31^ Furthermore, the importance of social mobilisation through NHWs as community mobilisers was demonstrated in India.^40^ Therefore, there is reason to believe Nigeria may benefit from these additional strategies in its vaccination campaigns. However, the cost-effectiveness and sustainability of using incentives for campaigns requires further evaluation.

### Strengths and Limitations

This is the first review to apply a realist approach to this complex question and the realist synthesis framework was particularly useful in enabling each intervention’s outcomes to be situated within its context. It generated realistic information on how and in what contexts strategies to improve childhood vaccinations work. Due to its focus on mechanisms of change, there was greater analytic power to explain the heterogeneity of results which enhances its transferability in terms of policy and practice. Also, it enabled gaps in knowledge and research to be identified.

However, certain limitations are noted. These reflect limitations to the studies that inform our conclusions and therefore the claims that can be made from them. Some articles did not provide sufficient detail to enable programme theories, mechanisms and contexts be fully surfaced. This limited the contributions of those articles to the synthesis. We also had relatively few studies from which to draw conclusions about some intervention categories. It is likely that older studies included in the synthesis will have been conducted in contexts that will have inevitably altered subsequently, and none will directly mirror the configuration of contextual factors at play in Nigeria. In the time since searches were conducted it is likely that new studies of relevance to the topic will have been published. Lastly, excluding non-English language studies may have resulted in some study selection bias. For these reasons we have been cautious when making recommendations based on the evidence synthesis. Specific evidence gaps identified include inconclusive results in relation to incentive-based strategies and health service integration studies. Also, a major question raised was on the cost-effectiveness and sustainability of the intervention programmes. We offer these as candidates for future research and evaluation.

## Conclusion

This is the first study to provide a realist review of the international evidence on strategies to improve childhood vaccination in LMICs. It provides configurations of context, mechanisms and outcomes as a means of informing vaccination coverage strategies in Nigeria. In line with the objectives of this study, interventions used in LMICs to improve vaccination access and uptake were identified and categorised as follows: communication/education, parental reminders, incentives, social mobilisation, health worker training and supportive supervision, health service integration and multi-pronged strategies. Communication strategies worked through different mechanisms at the individual and community level to produce results. These focused on developing the content, duration, settings of each educational session, utilising influential change agents, and monitoring and evaluation. The programme theories for the use of reminders, social mobilisation, staff training and supportive supervision were observed in practice, and these strategies were generally successful within study contexts. However, multi-pronged strategies drew on multiple or hybrid change theories and it is inherently difficult to identify which components (and the interactions between them) produce observed results. While the benefits and sustainability of incentives requires further study, it is clear that addressing the demand side of vaccination through incentives while leaving the supply side unattended may result in missed opportunities, suggesting the need to address both aspects. Finally, the implementation of health service integration would benefit from a more extensive evidence base.

This study provides additional information on strategies that can be utilised for further strengthening of RI and immunisation campaigns in hard-to reach, rural and urban communities in Nigeria. The findings from the study align with the current WHO/AFRO and National strategic plans for accelerating vaccination coverage,^3,10^ and provide additional insight to which strategies may be successful in various contexts. To the best of the authors’ knowledge, some of the interventions in other LMICs may not have been implemented in Nigeria, and some were implemented with different mechanisms. Therefore, this review provides information on what adjustments may be made to current programmes in order to produce better results.

## Ethical issues

Not applicable.

## Competing interests

Authors declare that they have no competing interests.

## Authors’ contributions

OSO and IW are both responsible for the study design. OSO performed the searches and led data extraction and analysis. IW checked data extraction and contributed to data analysis. OSO and IW both contributed to writing the manuscript.

## Supplementary files


Supplementary file 1 contains Table S1.
