# Supplementary material for: Realist Synthesis of the International Theory and Evidence on Strategies to Improve Childhood Vaccination in Low- and Middle-Income Countries: Developing Strategies for the Nigerian Healthcare System
Source: Int J Health Policy Manag. 2019 Dec 17;9(7):274–85. doi: 10.15171/ijhpm.2019.120 (PMC7444436; doi:10.15171/ijhpm.2019.120)
Supplement: Supplementary file 1 — contains Table S1. [file ijhpm-9-274-s001.pdf]

## Supplementary file 1

**Table S1.** General Characteristics of Studies Included in the Realist Synthesis

| **Source** | **Intervention type** | **Setting** | **Method** | **Participants** | **Outcomes** | **Conceptual richness** |
|---|---|---|---|---|---|---|
| Waisbord et al. 2010[31] | Communication: improving communication for polio eradication through real-time monitoring and evaluation. | Countries endemic to wild polio virus transmission: Afghanistan, India, Pakistan, and Nigeria | Qualitative evaluation of 4 polio-endemic countries | Individuals, households and communities | In India the number of unimmunised households, refusals and unreached communities reduced mostly through the impact of Community Mobilisation Coordinators (CMCs). In Pakistan and Afghanistan, there was a reduction in polio cases in nomadic populations and decreasing refusals for religious reasons. In Nigeria, communities no longer displayed block rejection of vaccination resulting in decreasing number of polio cases. | Thicker description |
| Oku et al. 2016[25] | Communication strategies: the communication interventions were directed at both Routine Immunisation (RI) and immunisation campaigns, to inform or educate; remind and recall; ensure acquisition of communication skills, enable communication and enhance community ownership | The study was conducted in 2 states in Nigeria- Bauchi state in Northern Nigeria and Cross River state in Southern Nigeria in their rural and urban settings. | Qualitative study, using semi-structured interviews, observations, and document analysis | Stakeholders involved in the development or delivery of vaccination communication strategies at the national, state and local government levels and also from development partners | Communication interventions were used less frequently in RI compared with immunisation campaigns due to funding allocation issues. Majority of the communication interventions were directed at informing and educating, and also remind and recall. Few interventions aimed to teach skills and enhance community ownership while no interventions aimed to provide support or facilitate decision-making in both states. There was lack of communication interventions directed at health workers. In Bauchi most interventions employed were campaigns, community based, polio-driven, and involved the use of appropriate channels to inform and educate mothers and community members. Also, caregivers were targeted at home in Bauchi. Fewer mobilisers were used to deliver communication interventions in Cross River, probably because this state has better vaccination coverage and because of the absence of polio. Also, enabling communications interventions were absent in Bauchi because one language is spoken universally, unlike in Cross River state. | Conceptually rich |

| Source | Intervention type | Setting | Method | Participants | Outcomes | Conceptual richness |
|---|---|---|---|---|---|---|
| Owais et al. 2011[26] | Communication strategies: through three targeted pictorial messages on vaccines lasting five minutes, taught by trained Community Health Workers (CHWs) to mothers at home, while the control group received 10-15 minutes general health education sessions. | Urban and semi-urban communities with low literacy and low immunisation coverage in Pakistan - Five low income sites | Multi-site community-based Randomised Controlled Trial (RCT) using educational intervention | 364 mother-infant pairs, with infants aged ≤ 6 weeks (Cut-off of 6 weeks was to ensure the intervention was implemented before the first dose of HBV/DPT was due) | There was a significant increase in DTP/HBV vaccine completion (3 doses) at four months after enrolment in the intervention group with almost three-quarters of the intervention group compared with only about half of the control group completing DPT3/HBV3. However, the study had a high refusal rate (27%) which may have excluded participants less likely to accept vaccines from the trial | Thicker description |
| Andersson et al. 2009[23] | Communication: evidence-based discussion on immunisation using three-phased discussion with community groups. The first discussion shared findings about vaccine uptake, the second focused on costs and benefits of vaccination, while the third focused on local action plans. | Lasbela, one of the poorest districts in Balochistan Province in Pakistan | Cluster RCT | 180 community groups in 18 clusters with each group having 8-10 participants, both male and female, and control group receiving usual care in 14 clusters | Measles and DPT vaccination uptake among children aged 12-23 months was significantly higher in intervention than in control clusters, where uptake fell over the intervention period. The intervention doubled the odds of measles vaccination in the intervention communities, and trebled the odds of full DPT vaccination. | Thicker description |
| Abdul Rahman et al. 2013[28] | Communication intervention: information campaign in 30 villages, utilising the help of peer spiritual leaders in half of them (Sorchi villages) but | Villages with low immunisation coverage in Akre district | A controlled before-and-after study | Villages with DPT 3 coverage rate <20% and a target | DPT3 and measles vaccination rates significantly increased while vaccine drop-out rates reduced post-intervention. Also, vaccination in Sorchi villages, where spiritual leaders were involved, improved significantly more than other villages | Thinner description |

| Source | Intervention type | Setting | Method | Participants | Outcomes | Conceptual richness |
|---|---|---|---|---|---|---|
| | not in the other half (non-Sorchi villages). | in Kurdistan region, Iraq | | population (aged<1year) | | |
| Pandey et al. 2007[27] | Communication: four to six public meetings were held in each intervention village cluster to disseminate information on entitled health services, education services, and village governance requirements | Village communities in Uttar Pradesh state, India | Cluster RCT | Households with at least 1 child going to public primary school in the village. Immunisation coverage targeted children aged 0-35 months | After 1 year, there were improvements in most services including 25% more infant vaccinations at intervention sites compared with control. Both low-caste and mid- to high-caste intervention households reported significant improvements in service delivery | Thinner description |
| Bolam et al. 1998[24] | Education: 20 minute, 1-to-1 health education (infant health promotion and family planning) sessions immediately after birth and/or 3 months later | Main government funded maternity hospital in Kathmandu, Nepal | RCT | 540 post-partum women | There were no other significant differences between intervention groups with regard to infant care or immunisation at 3 and 6 months of infants' age. However, there was an increased uptake of family planning. | Thinner description |
| Usman et al. 2011[30] | Communication and reminder: centre-based education of mothers on the benefits of completing immunisation, and risks of incomplete immunisation lasting 2-3 minutes and/or the use of redesigned, reminder-type immunisation cards | EPI centres in rural areas in Karachi, Pakistan | RCT | 1508 mother-child pairs visiting selected EPI centres for DTP1, who were resident in the study area for at least 6 months | Redesigned immunisation card alone, education to mothers alone, or both together were all effective in significantly increasing follow-up immunisation visits, evidenced by higher DPT3 completion rates, compared with control. Providing a redesigned card was more effective than education alone, and as effective as combination of both interventions. | Thinner description |
| Usman et al. 2009[29] | Communication and reminder: centre-based education of mothers on the benefits of completing | EPI centers in Urban areas of Karachi, Pakistan | RCT | 375 mothers with 1125 children registering for | Significant increase of 31% in DPT3 completion in the group that received both redesigned card and center-based education compared with the standard care group, while there was an increase of 25% and 18% in those | Thinner description |

| Source | Intervention type | Setting | Method | Participants | Outcomes | Conceptual richness |
|---|---|---|---|---|---|---|
| | immunisation, and risks of incomplete immunisation and/or the use of redesigned immunisation cards | | | DTP1 immunisation and residing in the study area for the past 6 months | that received the redesigned immunisation card or education respectively. | |
| Bangure et al. 2015[37] | Reminders: SMS reminders were sent at 6, 10 and 14 weeks in addition to routine health education. In the control group no SMS reminders were used, however routine health education was offered | Kadoma City clinics in Zimbabwe | RCT | Women who delivered babies, had cell phones and were residents of Kadoma City | Immunisation coverage was significantly higher in the intervention group than control, and there were reduced delays in the intervention group. Also, the intervention was cost-effective | Thicker description |
| Domek et al. 2016[38] | Reminders: SMS based vaccination reminder system | Two public health clinics in Guatemala City serving a publicly insured and low-income population | RCT (pilot study) | Infants aged 8–14 weeks presenting for the first dose of the primary immunisation series | Both intervention and usual care participants had high rates of vaccine completion, with a non-statistically significant higher percentage of children in the intervention completing both visits. The non-significant difference means that more research would be required to determine its effectiveness. More intervention parents agreed that SMS reminders would be helpful for remembering appointments, were interested in receiving future SMS reminders and willing to pay for future SMS reminders | Thicker description |

| Source | Intervention type | Setting | Method | Participants | Outcomes | Conceptual richness |
|---|---|---|---|---|---|---|
| Robertson et al. 2013[36] | Incentives: use of unconditional cash transfers (UCT) and conditional cash transfers (CCT) to improve child wellbeing using birth registration, vaccination uptake, and school attendance as indicators. | 12 sites in Manicaland, Zimbabwe, representing 4 socioeconomic strata: subsistence farming areas, roadside trading settlements, agricultural estates, and small towns | Matched Cluster RCT | Households within clusters, with children younger than 18 years, and some form of disabling household or child characteristic | The proportion of children aged 0–4 years with birth certificates had increased by 1·5% in the UCT group and by 16·4% in the CCT group by the end of the intervention period. The proportions of children aged 0–4 years with complete vaccination records was 3·1% greater in the UCT group and 1·8% greater in the CCT group than in the control group. Neither programme significantly increased the proportion of children with a complete vaccination record | Thicker description |
| Barham and Maluccio 2009[33]; Maluccio and Flores 2004[34] | Incentives: conditional cash transfer as part of a social safety net programme targeted at poor households living in rural Nicaragua | 42 localities in six poor rural municipalities in central Nicaragua | Cluster RCT | Households with children less than 5 years living within the target area | Immunisation coverage rates rose above 95% for DPT3 in the treatment group compared with 85% in the control group. There was an increase in on-time and catch-up vaccinations. The positive effects of the intervention were particularly large for hard to reach populations- less educated mothers or children living farther away from the health facility. Also, there was a substantial increase in vaccination coverage amongst controls. | Thicker description |
| Banerjee et al. 2010[32] | Incentives: A -once monthly reliable immunisation camp without incentive versus B - once monthly reliable immunisation camp with small incentives consisting of raw lentils and metal plates for completion of schedule versus control | Disadvantaged rural community with 2% immunisation coverage in Udiapur, India | Cluster RCT | Households with children less than 5 years old. 1640 children aged 0-6 months at baseline or 1-3 years at the endpoint survey were recruited | Following intervention, full immunisation rates were 39% for children receiving reliable immunisation with incentives, 18% for those receiving reliable immunisation without incentives, and 6% for control villages. Children in areas neighbouring intervention B villages were also more likely to be fully immunised than those from areas neighbouring intervention A villages. However, the rates are still too low to achieve herd immunity. Improving supply with incentives was more cost-effective than improving supply alone | Thicker description |

| Source | Intervention type | Setting | Method | Participants | Outcomes | Conceptual richness |
|---|---|---|---|---|---|---|
| Morris et al. 2004[35] | Incentives: household monetary incentives given to mothers who were either pregnant or had a child < 3 years of age to a maximum of 2 children versus service-level monetary incentive aimed at strengthening peripheral health services versus combination | 70 municipalities in rural Honduras | Cluster RCT | Households in 70 clusters including pregnant women, new mothers, and children aged < 3 years | The household-level package increased the coverage of first dose DTP/pentavalent vaccine administered at the appropriate age, but did not affect the coverage of immunisation against measles, or mothers' protection against tetanus. Based on mothers' reports, the household-level package had a marked impact on the uptake of antenatal care and routine well-child check-ups. Both of these indicators increased by 18–20 percentage points in the groups receiving the vouchers. There was no increase in use of services associated with the service-level package alone, and neither package affected uptake of the 10-day check-up. | Thicker description |
| Brugha and Kevany 1996[39] | Home visits by non-health workers (NHWs) | Urban setting with regular immunisation services in 3 towns in Ghana | Matched Cluster RCT | Children aged 12-18 months. Included 200 mother-and-child pairs in the intervention group and 219 in the control group | After 6 months, coverage had risen from 60% to 85%, which was 20% higher than in the control group in town 1. Also, the greatest benefit was in town 2 (which had the lowest pre-intervention coverage), where coverage rose from 21 (38.2%) to 50 (90.9%) through home immunisations. Little improvement was possible in town 3, where 37 of 39 children were already fully immunised before the intervention. Children were more likely to complete the schedule if their fathers were interviewed and participated in the decision to send them to the clinic. | Thicker description |

| Source | Intervention type | Setting | Method | Participants | Outcomes | Conceptual richness |
|---|---|---|---|---|---|---|
| Weiss et al. 2011[40] | Social mobilisation networks (SM Net) and Core Group Polio Project (CGPP) for polio eradication. | High risk districts in India - Western Uttar Pradesh and Central Bihar | Quasi-experimental uninterrupted time series analysis of secondary data | Villages in Community Mobilisation Coordinators (CMC) areas versus non-CMC areas | In each district, the mean booth coverage in CMC areas is higher than in non-CMC areas. In all districts except one (Meerut), the percent of X (unimmunised) households converted to P (immunised) was higher in CMC areas, on average, than in non-CMC areas. In all districts, the percent of X households converted to P during the second mop-up team activities was higher in CMC areas, on average, than in non-CMC areas. The vaccination outcomes in CGPP program areas met or exceeded the vaccination outcomes in non-program areas—even though program areas were purposively selected because the challenges vaccinating in these areas were greater. This is suggestive of the added-value of CGPP social mobilisation efforts. | Thicker description |
| Uskun et al. 2008[42] | Training of Primary Health Care workers to improve vaccination | Isparta city, which is a provincial centre in Southwest Turkey | Before and after study | 229 healthcare workers from primary health centres in Turkey | The intervention increased primary healthcare workers' knowledge about immunisation significantly. Gender, profession, content of training and performance of trainers were independent predictor variables for the difference between the pre- and post-workshop scores. After the intervention, a significant increase in vaccination coverage was seen in all vaccines in the national vaccination schedule | Thinner description |

| Source | Intervention type | Setting | Method | Participants | Outcomes | Conceptual richness |
|---|---|---|---|---|---|---|
| Djibuti et al. 2009[41] | Development of supportive supervision guidelines for district immunisation managers in 15 clusters | 67 districts in Georgia | Cluster RCT | District immunisation managers and PHC providers | Among immunisation managers, the intervention independently contributed to improved knowledge of supportive supervision, and helped remove self-perceived barriers to supportive supervision such as availability of resources to supervisors, lack of a clear format for providing supportive supervision, and lack of recognition among providers of the importance of supportive supervision. The intervention independently contributed to relative improvements in district-level service delivery outcomes such as vaccine wastage factors and the DPT-3 immunisation coverage rate. The clear positive improvement in all service delivery outcomes across both the intervention and control districts can be attributed to an overall improvement in the Georgian population's access to health care. | Thicker description |
| Briere et al. 2012[43] | Integration of immunisation with hygiene interventions such as free hygiene kits, education about water treatment and hand hygiene were given through group health talks (10–30 minutes) or one-on-one communication (5–15 minutes) | Homa Bay (intervention site) and Suba districts (comparison site), both largely rural districts, in Kenya | Quantitative evaluation using quasi-experimental design | Caregivers with a child aged 2-20 months | Significant increases were in reported household water treatment and correct hand washing technique in intervention households and no changes in comparison households. Immunisation coverage improved in both intervention and comparison infants. However, in the intervention site, there was no change in coverage among rural children, while in the comparison site, the same was true for urban children. Furthermore, vaccine coverage increased in rural areas of Suba in the absence of Hygiene kit distribution. Hence, the effect of the intervention on vaccination coverage is unclear. | Thicker description |

| Source | Intervention type | Setting | Method | Participants | Outcomes | Conceptual richness |
|---|---|---|---|---|---|---|
| Ryman et al. 2012[46] | Integration of IPT with EPI vaccination, a comparison of 2 strategies; using community workers to support nurses versus using nurses only. | Homa Bay district, largely rural district, in Kenya | Before and after qualitative and quantitative study | Caregivers with a child aged 2-20 months | Both nurse and community assisted strategies were well-accepted. Hygiene indicators improved similarly in nurse and community sites. Vaccination coverage increased in urban areas. In rural areas, coverage either remained unchanged or increased with the exception of OPV3 which decreased. Although both nurse and Safe Water and AIDS Project (SWAP) strategies were viewed favorably, additional advantages of the SWAP strategy were expressed, including the potential for SWAP members to have a slower and less intimidating educational style. | Thicker description |
| Dicko et al. 2011[44] | Integration of Intermittent preventive treatment (IPT) of malaria with EPI vaccines (DTP2, DTP3 and measles/yellow fever vaccine) in infants in 11 clusters. Control had standard care in 11 clusters | Kolokani, a district in Mali hyper endemic for malaria and with immunisation level < 50% | Cluster RCT | Children aged 0-23 months | At baseline, the proportion of completely vaccinated children was 36.7%, and rose 69.5% in the IPTi intervention zone compared to 53.8% in the non-intervention zone. The increase in vaccine coverage in the intervention zone compared to the non-intervention zone occurred for all the vaccines with the exception of BCG, the vaccine that was not given with IPTi. The proportion of children in the target age groups who received IPTi with DTP2, DTP3 and Measles, were 89.2%, 91.0% and 77.4% respectively compared to 2.3%, 2.6% and 1.7% in non-intervention zone. However, there was a significant increase in proportion of children who received vitamin A in each of the two zones post-intervention compared to baseline. Possible reasons for this include more adhesion of the community because of the high acceptability of the intervention, the additional training of the health workers and increase in supervision of EPI activities due to the introduction of IPTi-SP. | Thicker description |

| Source | Intervention type | Setting | Method | Participants | Outcomes | Conceptual richness |
|---|---|---|---|---|---|---|
| Mathanga et al. 2009[45] | Integration of Insecticide Treated Nets (ITN) with routine immunisation (RI) | Two rural districts in Malawi as intervention sites and a similar rural district as control | Before and after mixed methods (qualitative and quantitative) study | All pregnant women and children aged 12–23 months living in selected households | Insecticide-treated nets utilisation among children aged 12–23 months roughly doubled in the two intervention districts and did not change in the control district. Timely vaccination coverage increased in all three districts. The percentage of children aged 12–23 months who were both fully vaccinated by 12 months and slept under an ITN the night prior to the interview increased from 10–14% at baseline to 40–44% at follow-up in the intervention districts, but did not change significantly in the control district | Thicker description |
| Uddin et al. 2010[49] | Multi-pronged approach: Intervention package included: (a) an extended EPI service schedule; (b) training for service providers on valid doses and management of side-effects; (c) a screening tool to identify immunisation needs among clinic attendants; and (d) an EPI support group for social mobilization | Two Urban slums in Dhaka, Bangladesh | Before and after mixed methods study | Mothers of children aged 12–23 months, members of EPI support groups, and service providers. | 99% of the children were fully immunised post-intervention compared with only 43% before implementation. Antigen-wise coverage after implementation was also significantly higher compared with before implementation. Only 1% drop-out was observed after implementation of the interventions while it was 33% before implementation. At baseline, only 14% of children of working mothers were fully immunised compared with 75% of children of non-working mothers. However, post-intervention, 99% of children of working mothers were fully immunised, mostly attributed to the extended EPI service schedule. Although all of the interventions showed promise, the screening checklist to identify children with unmet need for immunisation identified a small number of children, suggesting this may not be as cost-effective as other interventions. | Conceptually rich |

| Source | Intervention type | Setting | Method | Participants | Outcomes | Conceptual richness |
|---|---|---|---|---|---|---|
| Hayford et al. 2014[47] | Multi-pronged approach: an intervention package which included: (a) an extended EPI service schedule; (b) training for service providers on valid doses and management of side-effects; (c) a screening tool to identify immunisation needs among clinic attendants; and (d) an EPI support group for social mobilisation | Two urban slums in Dhaka, Bangladesh | Qualitative evaluation and document review | 10 Key stakeholders - NGO clinic managers, vaccinators, other programme staff and Assistant health officer | The total economic cost of the 1-year intervention was $18,300, comprising external management and supervision (73%), training (11%), coordination costs (1%), uncompensated staff time and clinic costs (2%), and communications, supplies and other costs (13%). All interviewees believed the intervention was successful and recognised the dramatic impact it had on immunisation coverage. Key stakeholders ranked extended clinic hours and vaccinator training as the most important components of the intervention. External supervision was viewed as the most important factor for the intervention's success but also the costliest. Despite the perceived success and general satisfaction, none of the clinics have continued the pilot intervention because they reported they could not afford the additional supervisory salary, photocopying and meeting costs. In addition, they could not maintain the same level of supervision without an external supervisor or financial support. Two of the four clinics still maintained partially extended hours but could not stay open until 5 pm because the EPI ward room is no longer open after 5 pm. One NGO still used the target lists and no EPI Support Groups are active. Every manager and vaccinator reported they would do the intervention again, especially if additional funding were allocated. | Conceptually rich |

| Source | Intervention type | Setting | Method | Participants | Outcomes | Conceptual richness |
|---|---|---|---|---|---|---|
| Hu et al. 2015[48] | Multi-pronged approach: an immunisation package for migrant children which consists of: (1) extending the EPI service time and increasing the frequency of vaccination service; (2) training for vaccinators on valid doses and management of adverse events; (3) developing a screening tool to identify vaccination demands among migrant clinic attendants; (4) social mobilisation for immunisation. | Yiwu, a developed city in East China with rapid socio-economic growth | Before and after study and qualitative evaluation | 1548 migrant children aged 1-4 years, their mothers and vaccinators from 13 immunisation clinics. | The immunisation registration rate increased from 87.4 to 91.9 % after implementation of the EPI intervention package and the EPI card holding rate increased from 90.9 to 95.6 %. The coverage of fully immunised children increased from 71.5 to 88.6 % for migrant children aged 1–4 years. The correct response rates on valid doses and management of adverse events among vaccinators were over 90 % after training. The correct response rates on immunisation among mothers of migrant children were 86.8–99.3 % after interventions. The authors claim the EPI intervention package is sustainable because it was implemented within the existing vaccination service delivery system without any extra cost. Also, that vaccinators from the 13 immunisation clinics were willing to extend the EPI service time and increase the frequency of vaccination service without additional salaries, and the general practitioners in health facilities implemented the screening tool as a part of their ongoing work without any complain. Furthermore, that the training program for vaccinators was sustainable, because training for vaccinators was already a routine program of Yiwu CDC and the training materials on valid doses and management of AEFI had already been developed. | Thinner description |

| **Source** | **Intervention type** | **Setting** | **Method** | **Participants** | **Outcomes** | **Conceptual richness** |
|---|---|---|---|---|---|---|
| Uddin et al. 2012[50] | Multiple interventions: all four upazillas (subdivisions) received provider training and policy change regarding geographic boundaries. Then one upazilla in each district (Group A) was randomly chosen to receive two interventions (modified EPI immunisation session schedule and community support groups), and one upazilla (Group B) was chosen to receive a different intervention (screening checklist) | Rural hard-to-reach areas of Bangladesh: 4 subdistricts in Sylhet (a hoar area that remains under water almost for the whole year) and Chittagong (a hilly area) | Pre- and post-intervention survey and qualitative evaluation | Mothers of children aged 12-23 months, service providers, and community support group members. | The valid coverage increased at endline compared to baseline in the study areas, and the difference of the increase was highly significant. The findings also showed that the number of drop-outs, left-outs, and invalid doses decreased at endline compared to baseline in the study areas, and the difference was also highly significant. The immunisation coverage improved significantly in all the four study sub-districts that received interventions, although the relative contribution of each intervention is unknown. Results from regression analysis shows that use of screening checklist was more effective in improving child immunisation coverage than modified EPI session schedule and community support group in some areas. The service providers confirmed that the screening checklist was user-friendly and useful to identify and address the missed opportunity for immunisation. These findings support the inclusion of the screening checklist in the EPI, especially in the low-performing areas. It is suggested that the programme is both scalable and sustainable because the package of interventions was implemented within the existing local health system by the staff members of the Ministry of Health and Family Welfare at no extra cost, and refresher training is scalable since the training materials are already available. However, the sustainability of the support groups is doubtful because of the lack of financial incentives. | Conceptually rich |
